# Erythema ab igne—A Potential Cutaneous Marker of Chronic Heat Use in Patients with Endometriosis: A Narrative Literature Review and a Case Report

**DOI:** 10.3390/life15101533

**Published:** 2025-09-29

**Authors:** Francesca Scurtu, Lucian G. Scurtu, Alexandra Irma Gabriela Baușic, Aida Petca, Claudia Mehedințu

**Affiliations:** 1 Department of Obstetrics and Gynecology, “Carol Davila” University of Medicine and Pharmacy, 050474 Bucharest, Romania; francesca.scurtu@umfcd.ro (F.S.); alexandra.bausic@umfcd.ro (A.I.G.B.); aida.petca@umfcd.ro (A.P.); claudia.mehedintu@umfcd.ro (C.M.); 2 Department of Obstetrics and Gynecology, Filantropia Clinical Hospital, 030084 Bucharest, Romania; 3 Department of Dermatology I, Colentina Clinical Hospital, “Carol Davila” University of Medicine and Pharmacy, 020125 Bucharest, Romania; 4 Department of Obstetrics and Gynecology, “Prof. Dr. Panait Sîrbu” Obstetrics and Gynecology Hospital, 060251 Bucharest, Romania; 5 Department of Obstetrics and Gynecology, Elias University Emergency Hospital, 011461 Bucharest, Romania

**Keywords:** endometriosis, erythema ab igne, toasted skin syndrome, hot bottle rash, squamous cell carcinoma, Marjolin ulcer, heating device, pain, deep endometriosis, laparoscopy

## Abstract

Erythema ab igne (EAI), also known as “hot water bottle rash” or “toasted skin syndrome”, is a benign cutaneous condition caused by chronic exposure to low-level infrared heat. It typically begins as transient erythema and evolves into a reticulated brown pigmentation with telangiectasias. A skin biopsy, ideally taken from the central area of the hyperpigmented lesion, is recommended to exclude differential diagnoses. Although usually benign, EAI has been associated with rare malignant transformations, supported only by low-level evidence. Elimination of the heat source is essential, and topical treatments such as hydroquinone or retinoids may be considered, while agents like 5-fluorouracil or imiquimod are reserved for dysplastic lesions. Women with endometriosis frequently use heating devices to alleviate dysmenorrhea and chronic pelvic pain. However, prolonged or inappropriate heat application can lead to chronic thermal injury, including EAI, and may delay medical consultation. While controlled trials confirm short-term analgesic efficacy of heat therapy, extrapolating these findings to unrestricted home use without standardized safety recommendations can be misleading. EAI illustrates the broader impact of chronic pain in endometriosis, linking cutaneous manifestations with neuroplastic alterations and psychiatric comorbidities. A nuanced approach combining patient education on safe use of heat, close dermatologic monitoring, and multidisciplinary pain management is warranted.

## 1. Introduction

Erythema ab igne (EAI) represents a benign skin finding secondary to chronic exposure to low levels of external infrared heat, typically around but below the burn threshold (43–47 °C). EAI was previously described as the “hot water bottle rash” or “toasted skin syndrome,” as it typically appeared on the pretibial skin of elderly individuals due to the use of space heaters. Nowadays, the main triggers are resting laptops for a prolonged time (professional EAI) and using heating devices to alleviate chronic pain. Chronic EAI has the potential to evolve into skin cancer [1].

Chronic pelvic pain (CPP) is recurrent or persistent pelvic pain that lasts at least six months [2]. It is encountered in up to 25% of menstruating persons worldwide. Between 40 and 87% of CPP patients have endometriosis. Endometriosis arises as estrogen-dependent and progesterone-resistant extrauterine lesions that induce a chronic inflammation, provoking pain and infertility. Endometriosis-associated pain usually debuts after menarche and is secondary to retrograde menstruation and neuro-angiogenic factors that activate endometrial cells into developing endometrial lesions [3]. Up to 37% of patients with pelvic endometriosis develop deep endometriosis (DE), involving the Douglas pouch, the uterosacral ligaments, the posterior vaginal wall, the colorectal tract, and the urinary tract. Severe pain (dysmenorrhea, deep dyspareunia, intermenstrual pelvic pain) is encountered in more than 90% of DE patients [4].

The 2022 ESHRE Guidelines showed that non-medical management strategies, including heating devices, are widely used by patients with endometriosis to alleviate severe pain. Still, EAI is not traditionally linked to endometriosis [5], and the literature is scarce on the topic. Considering the potential risk of neoplastic transformation associated with EAI, this article aims to raise awareness among both gynecologists, who are frequently faced with the diagnosis of endometriosis, and dermatologists, who may encounter EAI lesions, as well as among patients themselves, to help identify and prevent harmful, extreme behaviors driven by intense pain. This manuscript provides a literature review on the subject alongside a clinical case from our practice.

## 2. Materials and Methods

A comprehensive literature search was carried out using PubMed (MEDLINE), Scopus, and Google Scholar to explore the clinical and pathophysiological intersections between EAI and endometriosis. The search strategy combined the following key terms and their variations: “erythema ab igne”, “hot water bottle rash”, “toasted skin syndrome”, “endometriosis”, “dysmenorrhea”, “dyspareunia”, “behavior”, and “chronic pelvic pain.” Boolean operators (AND/OR) were applied to capture studies linking heat-related skin changes with gynecological pain syndromes. The search covered the period from January 1992 to 31 July 2025, which represents the date of the last update.

Eligible sources were peer-reviewed articles published in English that discussed erythema ab igne or related cutaneous effects of heat, endometriosis and its associated pain syndromes, behavioral coping strategies such as heat therapy, or relevant psychological and dermatological aspects. We excluded non-peer-reviewed publications, such as conference abstracts without full text, editorials, or letters lacking clinical data, as well as articles not available in English or papers unrelated to either endometriosis or EAI.

The available literature proved to be extremely scarce. This limitation made a systematic methodology unfeasible, and therefore, the work was structured as a narrative review, complemented by the presentation of a representative clinical case. No formal quality assessment tool was applied, but priority was given to studies published in peer-reviewed journals, clinical guidelines from professional societies, and recent high-quality reviews whenever possible.

## 3. Results

### 3.1. EAI—Definition and Etiopathogenesis

EAI is a benign skin rash characterized by asymptomatic, reticulated, brown macules caused by chronic exposure to external heat sources, below the skin burn threshold [1]. The duration between heat exposure and EAI is largely variable (weeks to years). EAI is more frequent in women, with a 10:1 sex ratio. The average age of EAI is 28.6 +/− 10.4 years [1,6]. Radiant heaters (surface temperature usually 43–50 °C, depending on settings and distance), laptops (surface temperature up to 40–47 °C, often modified by clothing acting as a barrier), hot water bottles (typically 43–50 °C if applied directly, lower when wrapped in towels or fabric covers), heating pads (typically 43–50 °C if applied directly, lower when wrapped in towels or fabric covers), and heated car seats (typically 43–50 °C if applied directly, lower when wrapped in towels or fabric covers) are frequently incriminated [7,8,9]. Recently, virtual reality headset-induced EAI has been described [10]. EAI may be considered an occupational dermatosis in glass blowers, jewelers, bakers, workers in the metallurgy industry, chefs, and tandoor oven users [6,8,11].

EAI can sometimes point to an internal malignancy, as shown by Bunick et al., including pancreatic adenocarcinoma, gastric adenocarcinoma, colorectal adenocarcinoma, hepatic metastases, spinal metastases, and lymphoma [12]. Hence, internal malignancies should be ruled out in any patient with CPP and EAI.

### 3.2. Clinical Appearance and Differentials of EAI

EAI initially presents as a transitory erythema and later progresses into a brown, reticular pattern with telangiectasias [9]. The most important differential diagnosis of EAI is *livedo reticularis* (LR). LR is classified into physiologic LR (*livedo marmorata*) and pathologic LR. *Livedo marmorata* represents a normal vasospastic response, appearing upon cold exposure and disappearing upon local warming. In contrast, pathologic LR is persistent [13].

Table 1 outlines the main differential diagnoses of EAI, including conditions associated with pathologic LR [6,13,14,15,16]. Rare forms of bullous EAI have been described in patients with diabetes mellitus, anemia, eating disorders, and hypothyroidism. In this case, bullous diseases, such as bullous pemphigoid and bullous lupus erythematosus, should be ruled out [6].

### 3.3. Pathology of EAI

Since the pigmented, lacy pattern lesions signify local venous congestion, and the white, central area is represented by inflamed or obstructed arterioles, the skin fragment should always be obtained from the central area and not from the rings themselves [13]. The exact pathophysiology of EAI is unknown, but pathology samples display both epidermal and dermal alterations. In the early stages of EAI, the epidermis is atrophic, with scarce atypical keratinocytes; vasodilatation, perivascular inflammation, and hemosiderin deposits are seen in the dermis. In mature EAI lesions, the epidermis displays dyskeratosis, focal parakeratosis, and vacuolization. Still, the usual histopathology findings are nonspecific and exclude other diagnoses [6,17].

A subepidermal blister is encountered in bullous EAI. Direct immunofluorescence (DIF) should be performed to exclude other subepidermal bullous disorders. In the setting of a non-concluding pathology report, DIF may also exclude vasculitis (Table 2) [18,19].

### 3.4. EAI-Associated Malignization

Later stages of EAI can demonstrate increased dyskeratosis and dermal elastosis and pose the risk of malignant transformation [20]. Unlike the intensely studied UV radiation, the biological effects of infrared radiation (IRR) on human skin are less studied. The carcinogenic effect of IRR was described by decreasing keratinocyte apoptosis and promoting proliferation via heat shock proteins (HSPs). Temperatures above 39 °C (heat shock), but below 45 °C, trigger the activation of HSPs, intracellular and ubiquitary proteins responsible for the correct folding and transport of other proteins, as well as the reassembly of proteins that have been misfolded as a result of heat stress [21,22].

The heat shock response consists of trimerization and phosphorylation of heat shock factor (HSF), which translocates into the nucleus and promotes the expression of HSPs (especially HSP70 and HSP92). HSPs determine defective DNA replication, transcription, and repair. HSPs activate NRAS and BRAF kinases and promote keratinocyte proliferation. Additionally, it inhibits the keratinocytes’ apoptosis by down-regulating death signaling pathways, such as the p53 protein and c-Jun NH_2_ terminal kinase (JNK) [21,22,23,24,25].

**Figure 1:**
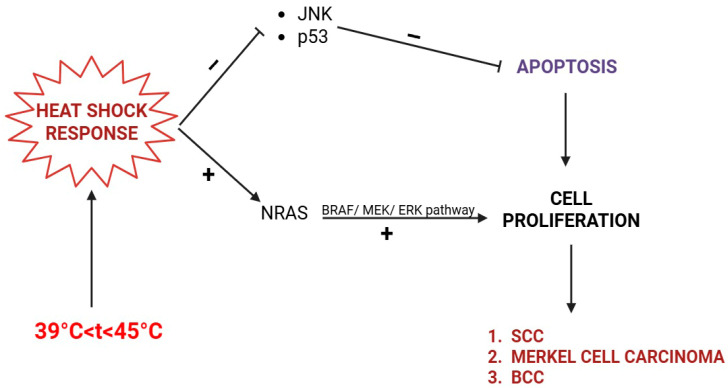
Skin cancers (BCC, SCC, Merkel cell carcinoma) in EAI induced by infrared radiation (IRR). The heat shock response inhibits apoptosis (via JNK and p53) and stimulates cell proliferation via the NRAS/BRAF/MEK/ERK pathway.

Hence, although EAI is a benign skin condition, it may eventually evolve into a squamous cell carcinoma (SCC), Merkel cell carcinoma, or even basal cell carcinoma (BCC) (Figure 1). For this particular reason, a long-term dermatological follow-up is generally recommended in EAI patients, and any ulceration should be promptly biopsied [6,26], as it may represent a Marjolin ulcer. Marjolin ulcer represents an aggressive SCC arising within a preexisting skin disorder, usually chronic inflammation or scarring [27].

### 3.5. EAI Treatment

The first-line treatment for EAI is the elimination of the IRR source. However, the reticular, hyperpigmented pattern may persist indefinitely or slowly fade [6]. Topical treatments such as hydroquinone and retinoids can help improve the appearance of the skin. Additionally, the application of 5-fluorouracil (5-FU) and imiquimod may be beneficial if keratinocyte dysplasia is present [28,29,30].

Kim et al. obtained good cosmetic results using a 1064 nm low-fluence Q-switched Neodymium-Doped Yttrium Aluminum Garnet (QS Nd-YAG) laser at weekly intervals in a patient with EAI and no evidence of dysplasia [31]. SCC secondary to EAI should be surgically excised, and the patients may benefit from a Mohs micrographic surgery approach [32].

### 3.6. Endometriosis-Related EAI

Dysmenorrhea is the central clinical feature of endometriosis. However, it is divided into primary dysmenorrhea, which occurs in the first 6–12 months after menarche, and secondary dysmenorrhea, which occurs 12 months after menarche [33]. It is estimated that dysmenorrhea affects around 70–93% of women [34]. The pain mechanism occurs through increased production of prostaglandins, leukotrienes, and vasopressin, which induce increased myometrial contractility and vasoconstriction, resulting in endometrial ischemia [35,36,37].

Endometriosis and adenomyosis are the main causes of secondary dysmenorrhea, which gradually transforms into acyclic pain and chronic pelvic pain [35,38]. Endometriosis often causes symptoms that fluctuate with the menstrual cycle, varying from mild to severe. These can include pain in the abdomen, lumbosacral region, perineum, buttocks, rectum, and vulvovaginal area [39]. Some patients may also experience weakness, dyspareunia, and loss of bladder or bowel control [40]. When the central or peripheral nervous system is involved, neurological symptoms such as leg and pelvic pain, cyclic radiculopathy of the lower limbs, urinary incontinence, and, in rare cases, paraplegia can occur [41]. Nerve involvement plays a key role in endometriosis-related pain. Ectopic endometrial tissue can irritate or invade peripheral nerves, triggering pain [42].

Neuroangiogenesis (new blood vessel growth around nerves) promotes inflammation and nerve activation, contributing to perineural invasion [43]. Both peripheral and central sensitization occur, heightening pain perception. Additionally, adhesions may compress nerves, causing sharp, persistent pain and hyperalgesia [44]. Severe pain often suggests direct nerve involvement. Endometriosis can also present with gastrointestinal and urinary symptoms like constipation, diarrhea, or dysuria, which may complicate diagnosis [45,46]. These factors underscore the complex pain pathways involved and the importance of multidisciplinary care.

Common CPP causes include inflammatory bowel disease, nerve entrapment, adenomyosis, endometriosis, adhesions, cystitis, internal malignancies, chronic endometritis, vulvodynia, and musculoskeletal etiologies. The Carnett test differentiates between visceral and abdominal wall pain. Additionally, depression and trauma history should be carefully investigated [47].

Heating pads alleviate pain among 29.5% of patients who experience CPP [48]. A Canadian study showed that alternative therapies, especially heat and cannabis, are commonly used by patients with endometriosis [49]. Women with endometriosis use an average of 5.8 self-care interventions more than once a week, including heat, rest, over-the-counter pain medications, and diet changes [50]. However, according to the 2022 ESHRE Guidelines, no recommendations can be made regarding non-medical interventions to reduce pain, including nutrition, electrotherapy, acupuncture, physiotherapy, exercise, and psychological interventions [8].

CPP is increasingly recognized as more than a physical disorder, as its persistence induces neuroplastic changes in the central nervous system that predispose to psychiatric comorbidities, particularly within the anxiety–depressive spectrum [51]. Primary dysmenorrhea has been associated with a 1.7-fold increased risk of depressive disorders [52], and women with CPP, often overlapping with endometriosis, show high rates of anxiety and depression (up to 48%), closely related to pain severity rather than disease stage [53,54]. Systematic reviews confirm that chronic visceral pain almost invariably coexists with psychiatric symptoms [55,56]. These psychological burdens may encourage maladaptive coping behaviors, including the excessive use of heat therapy, which underscores the importance of integrating dermatologic risk awareness and patient education into comprehensive pain management.

Regarding the alleviation of dysmenorrhea, it is essential to understand and educate patients on the appropriate and safe use of heat-based devices specifically designed for this purpose. While local heat application is known to provide significant symptomatic relief by improving pelvic blood flow, reducing uterine contractility, and modulating pain perception, it is not without potential risks. Prolonged or inappropriate use of heating pads can lead to skin burns or chronic thermal injury, including EAI. This is particularly relevant in patients with endometriosis, who often rely on long-term and recurrent heat therapy due to persistent pelvic pain. Misuse of such devices may not only provoke skin damage but also delay medical consultation or mask the progression of underlying disease. Unfortunately, some publications extrapolate data from clinical studies and oversimplify it in public-facing articles, claiming that heat application has no adverse health consequences [57].

This is misleading, as the cited studies typically use heat therapy in a strictly controlled setting, standardized in terms of temperature, duration, and application method, often within randomized controlled trials designed to assess short-term analgesic efficacy [58,59]. Translating these findings into blanket statements for general use, without discussing safety parameters, may encourage inappropriate or excessive use, particularly in vulnerable populations such as women with endometriosis.

The management of EAI in patients with endometriosis does not differ from standard treatment protocols. However, the chronic nature of EAI and the patient’s frequent willingness to conceive following DE surgery may complicate the management of EAI in newly pregnant patients. In this context, topical therapies with hydroquinone, retinoids, imiquimod, and 5-FU are generally not recommended [60], and a wait-and-see attitude is preferable.

Given the strong link between chronic pelvic pain, maladaptive coping behaviors such as excessive heat use, and the risk of EAI, we propose a practical clinical algorithm for screening and management (Table 3).

### 3.7. Evidence Gaps

Current knowledge on EAI in endometriosis is limited. The prevalence of EAI in this population remains unreported, and the overall cancer risk is undefined and based only on low-level evidence. No standardized safety recommendations exist for the use of home heat devices in chronic pelvic pain. Addressing these gaps requires larger observational studies and the development of clear safety guidelines.

## 4. Case Presentation

A 33-year-old nulliparous Caucasian woman was referred with a prolonged history of diffuse abdominal and pelvic pain, dysmenorrhea, and deep dyspareunia. She described dysmenorrhea beginning at the age of 18 years old, approximately four years after the menarche, with a progressive worsening over the past decade. Two years before presentation, she developed CPP with a distinct cyclical pattern: discomfort commenced around the time of ovulation and progressively worsened until the onset of menstruation, during which it peaked in intensity. The pain was described as dull, bilateral, and cramp-like, often radiating to the lower back and inner thighs. Despite regular use of NSAIDs, symptomatic relief was inconsistent, and due to the refractory nature of her symptoms, the patient adopted extreme thermal measures, including frequent hot showers and continuous application of heating pads, particularly during menstruation.

She also reported deep dyspareunia with penetrative intercourse, which significantly impacted her sexual activity and quality of life. She denied gastrointestinal and urinary symptoms, including dyschezia or hematuria, and had no prior history of pelvic inflammatory disease or surgery. Her menstrual cycles were regular (28–30 days) and lasted approximately 5–6 days. She had been using barrier contraception and never tried to conceive. There was no significant family history of endometriosis or gynecologic malignancies.

**Figure 2:**
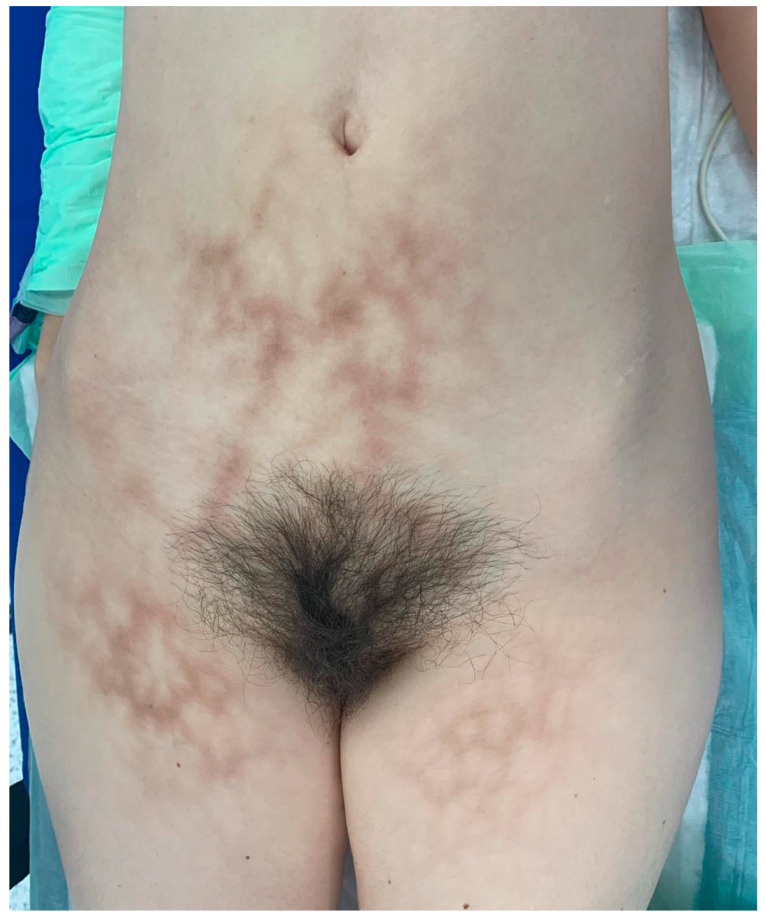
Reticular, brown, symmetric macular exanthema involving the anterior lower abdomen and superior thighs.

The clinical examination revealed diffuse, reticulated hyperpigmentation of the lower abdomen and upper thighs concerning *livedo reticularis*. Nodules and ulcerations were absent (Figure 2). The skin rash was not symptomatic. She first noticed the eruption 10 months before hospital admission, concomitant with the use of heating pads, with a distribution corresponding to the heating devices. The patient used the heating pads for 4 to 5 h daily and sometimes during nighttime sleep.

The patient underwent a gynecological evaluation and a pelvic ultrasound with a suspected diagnosis of DE. Abdominal palpation revealed mild tenderness in the lower abdomen. Pelvic examination demonstrated uterine tenderness with limited mobility and nodularity along the uterosacral ligaments. Pain was reproduced upon deep vaginal palpation and cervical motion. A transvaginal ultrasound (TVUS) examination demonstrated a subtle hypoechoic area along the uterosacral ligaments. Furthermore, an irregular hypoechoic nodule with ill-defined margins was detected on the anterior rectal wall, indicative of DE. The absence of a sliding sign between the posterior uterine wall and the anterior rectum suggests the presence of adhesions and DE.

Pelvic MRI revealed diffuse adenomyosis. A conglomerate of multiple right ovarian endometriomas (*n* = 7) was identified, the largest located on the posterior aspect of the ovary. Four left ovarian endometriomas were described, with the largest situated at the posterior pole; the remaining lesions were infracentrimetric. Bilateral superficial periovarian endometriotic implants were also noted. A deep endometriotic lesion involving the posterior uterine wall was observed at the insertion of the uterosacral ligaments and in the retrocervical region, adherent to the posterior vaginal fornix and the mid-rectum. Endometriotic infiltration was detected along the course of the uterosacral ligaments with parametrial extension and bilateral ovarian adhesions, more pronounced on the right side. A “kissing ovaries” appearance was present. Superficial infiltration of the mid-rectal wall was described at the site of uterine adhesion, approximately 8 cm cranial to the anal verge, without invasion of the *muscularis propria*. The pouch of Douglas appeared obliterated due to an adhesive complex involving the uterus, rectum, and both ovaries.

Preoperatively, a screening for connective tissue disorders excluded systemic lupus erythematosus, ANCA vasculitis, and anti-phospholipid antibody syndrome. The dermatology evaluation suspected EAI, given the patient’s exposure to external localized heat. A 6 mm punch biopsy was performed to exclude differentials. The pathology report revealed a thin epidermis, non-specific perivascular infiltrate (hematoxylin–eosin stain), and fragmented elastic fibers (Van-Gieson stain), consistent with the diagnosis of EAI.

**Figure 3:**
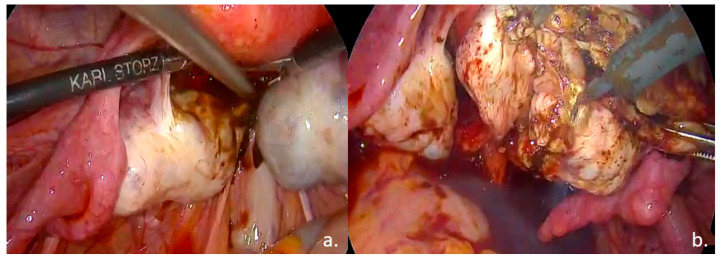
Intraoperative findings: (**a**). Bilateral ovarian endometriomas; (**b**). ovarian cystectomy.

**Figure 4:**
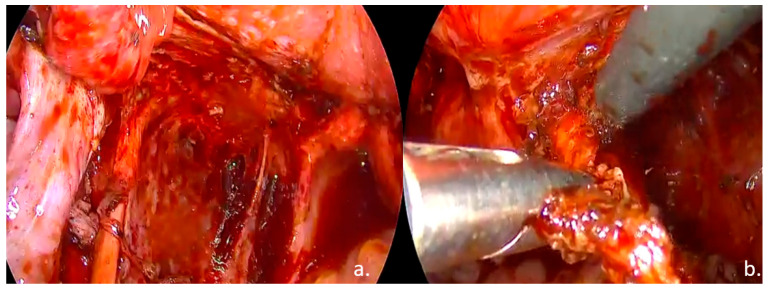
Intraoperative findings: (**a**). left ureterolysis, excision of the left ovarian fossa peritoneum; (**b**). Excision of the left uterosacral ligament.

**Figure 5:**
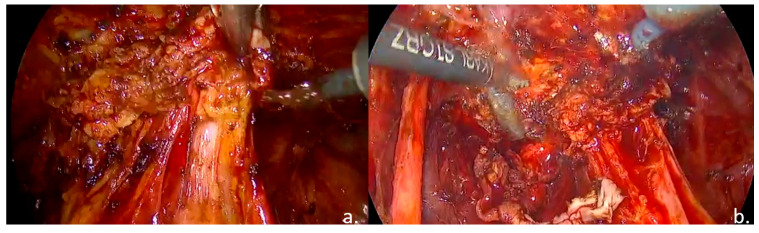
Intraoperative findings: (**a**). nodule of the rectovaginal septum and rectum; (**b**). rectal shaving.

Laparoscopic surgical treatment included extensive pelvic adhesiolysis and restoration of normal pelvic anatomy. Complete excision of endometriotic lesions was performed at the level of the uterosacral ligaments, rectovaginal septum, and posterior vaginal fornix, and a rectal shaving procedure was carried out. Bilateral ovarian cystectomy with excision of endometriomas was performed. The postoperative #ENZIAN classification was A2B2C2FA, indicating moderate-to-severe involvement of the retrocervical/vaginal compartment (A2), uterosacral ligaments/parametrium (B2), and rectum (C2), along with bilateral ovarian involvement and associated adenomyosis (F: ovaries and uterus) (Figure 3, Figure 4 and Figure 5).

The patient was advised to discontinue the heating pads, and the pigmentation ameliorated under hydroquinone and retinoid topical treatments. Abdominal and pelvic pain, dysmenorrhea, and dyspareunia were solved postoperatively. Regular dermatology follow-up was recommended for the patient.

## 5. Conclusions

While EAI remains a benign skin condition resulting from heat exposure, it possesses the potential for neoplastic transformation by disrupting normal apoptotic processes and promoting cell proliferation, thereby increasing the risk of non-melanoma skin cancers (SCC, Merkel cell carcinoma, BCC). Differential diagnoses of EAI are complex, and although the diagnosis is generally clinically based, a skin biopsy from the center of the ring will certainly rule out differentials, such as LR or vasculitis. A bullous EAI should be examined additionally with a DIF.

This review particularly emphasizes the vulnerable subgroup of patients suffering from dysmenorrhea and chronic pain due to endometriosis, who frequently seek both conventional and alternative therapies for symptomatic relief. EAI in these patients represents more than a dermatological condition: it symbolizes the severe pain that follows these patients throughout their daily lives and their somatic disability.

As the recognition of endometriosis has grown over the past decade, it is crucial to implement new preventive strategies, promote accurate dissemination of information, and raise awareness about the risks associated with heat exposure. A more nuanced understanding of heating devices is required, combining patient education on safe usage with a multidisciplinary approach to endometriosis-associated pain management. Healthcare professionals, including gynecologists, dermatologists, and family physicians, must play an active role in patient education, risk assessment, and management, ensuring that patients receive care with the utmost respect and understanding of the potential dangers.

## Figures and Tables

**Table 1:** Differential diagnoses of erythema ab igne (EAI).

Etiology	Conditions
Hematologic/Hypercoagulability disorder	Antiphospholipid antibody syndrome Cryopathies (cryoglobulinemia, cryofibrinogenemia, cold agglutinins) Polycythemia vera Essential thrombocytosis Protein C and protein S deficiency Deep venous thrombosis Thrombocytopathy
Autoimmune diseases/vasculitis	SLE Rheumatoid arthritis Sjogren’s syndrome SS Dermatomyositis Still’s disease Sharp’s syndrome Small and medium vessel vasculitis Polyarteritis nodosa Fibromyalgia
Vessel wall disorders/emboli	Calciphylaxis Septic emboli, cholesterol emboli Atrial myxoma
Infections	Hepatitis C Mycoplasma pneumonia Brucella, Rickettsia Tuberculosis Meningococcemia Syphilis Endocarditis SARS-CoV-2 Parvovirus B-19
Neurological disorders	Diabetic neuropathy Parkinson’s disease Stroke Multiple sclerosis Migraine Reflex sympathetic dystrophy
Neoplasia	Renal cell carcinoma Metastatic breast cancer
Lymphoma/leukemia	Mycosis fungoides Angiotrophic lymphoma Acute lymphocytic leukemia Chronic natural killer cell leukemia Multiple myeloma
Medications	Amantadine Minocycline Gemcitabine Presors (ergotamine, cocaine) Interferons (alpha and beta) Heparin Combination of antiretroviral (lopinavir) and antipsychotics (aripiprazole)
Others	Anorexia nervosa Physical abuse Chronic pancreatitis Homocysteinuria Poikiloderma atrophicans vascularis Panniculitis

**Table 2:** Direct immunofluorescence (DIF) differential diagnoses of bullous EAI.

Etiology	Direct Immunofluorescence (DIF)
Pemphigus vulgaris	Intercellular space deposits of C3, IgG (chicken wire pattern)
Pemphigus paraneoplastic	Intercellular space and basement membrane zone deposits of C3, IgG
Bullous Pemphigoid	Basement membrane zone linear deposits of C3 > IgG
Bullous lupus erythematosus	Granular and/or linear deposits of IgG and C3 +/− IgA or IgM along the basement membrane zone (lupus band)
Dermatitis herpetiformis	Granular IgA deposits along the basement membrane zone and within dermal papillae
Leukocytoclastic vasculitis	Granular/fibrillar deposits of IgA > IgG/IgM, C3, or fibrinogen within the walls of superficial small vessels
Bullous erythema ab igne	Negative

**Table 3:** Clinical algorithm for screening and management of EAI in endometriosis patients.

**Screening and clinical suspicion**	Identify patients at risk	- Endometriosis/CPP patients using heating devices (hot water bottles, pads, heated seats) - History: device type, temperature, frequency, barriers (clothing/towel) - Assess coping behaviors, pain severity, psychiatric comorbidities - Physical exam: reticulated brown macules/telangiectasias; urgent referral if ulcerated
**Diagnostic work-up**	Confirm diagnosis and rule out differentials	- Clinical diagnosis in typical cases - Biopsy from central lesion if atypical/ulcerated/non-healing - DIF (if bullous) to exclude autoimmune blistering - Laboratory/imaging if suspected vasculitis/internal malignancy
**Management**	General, dermatologic, and gynecologic care	General: - Eliminate/reduce heat exposure - Psychological support for coping Dermatologic: - Hydroquinone, retinoids for pigmentation (not in pregnancy) - 5-FU or imiquimod if dysplasia - Q-switched Nd-YAG laser for persistent lesions Surgical excision/Mohs if malignant Gynecologic/Pain: - Optimize endometriosis treatment (hormonal/surgical) - Non-thermal coping: medication, physiotherapy, cognitive behavioral therapy, lifestyle
**Follow-up**	Ongoing multidisciplinary care	- Dermatology: every 6–12 months, earlier if ulceration - Gynecology: ensure pain control to prevent maladaptive heat use - Psychological: address anxiety/depression, reinforce safe coping

## Data Availability

No new data were created or analyzed in this study.

## References

[1] Cabrera Hernández A., Beà Ardebol S., Medina Montalvo S., Trasobares Marugán L. (2016). Eritema ab igne. Reumatol. Clin..

[2] Aredo J.V., Heyrana K.J., Karp B.I., Shah J.P., Stratton P. (2017). Relating Chronic Pelvic Pain and Endometriosis to Signs of Sensitization and Myofascial Pain and Dysfunction. Semin. Reprod. Med..

[3] Karp B.I., Stratton P. (2023). Endometriosis-associated chronic pelvic pain. Med.

[4] Mehedintu C., Frincu F., Brinduse L.A., Carp-Veliscu A., Bratila E., Hennetier C., Roman H. (2021). Postoperative Assessment of the Quality of Life in Patients with Colorectal Endometriosis. J. Clin. Med..

[5] Becker C.M., Bokor A., Heikinheimo O., Horne A., Jansen F., Kiesel L., King K., Kvaskoff M., Nap A., Petersen K. (2022). ESHRE guideline: Endometriosis. Hum. Reprod. Open.

[6] Ozturk M., An I. (2020). Clinical features and etiology of patients with erythema ab igne: A retrospective multicenter study. J. Cosmet. Dermatol..

[7] Gmuca S., Yu J., Weiss P.F., Treat J.R., Sherry D.D. (2020). Erythema Ab Igne in an Adolescent with Chronic Pain: An Alarming Cutaneous Eruption From Heat Exposure. Pediatr. Emerg. Care.

[8] Baig M.N., Byrne F. (2018). Erythema Ab Igne and Its Relation to Spinal Pathology. Cureus.

[9] Kesty K., Feldman S.R. (2014). Erythema ab igne: Evolving technology, evolving presentation. Dermatol Online J..

[10] Moreau T., Benzaquen M., Gueissaz F. (2022). Erythema ab igne after using a virtual reality headset: A new phenomenon to know. J. Eur. Acad. Dermatol. Venereol..

[11] Ashka S., Dhvani C., Sejal T., Raksha P. (2021). An Unusual Presentation Of Erythema Ab Igne And The Role Of Occupational History In Unveiling It—A Case Report. Natl. J. Integr. Res. Med..

[12] Bunick C.G., King B.A., Ibrahim O. (2014). When erythema ab igne warrants an evaluation for internal malignancy. Int. J. Dermatol..

[13] Rose A.E., Sagger V., Boyd K.P., Patel R.R., McLellan B. (2013). Livedo reticularis. Dermatol. Online J..

[14] Dean S.M. (2011). Livedo reticularis and related disorders. Curr. Treat. Options Cardiovasc. Med..

[15] Sajjan V.V., Lunge S., Swamy M.B., Pandit A.M. (2015). Livedo reticularis: A review of the literature. Indian Dermatol. Online J..

[16] Khalil S., Hinds B.R., Manalo I.F., Vargas I.M., Mallela S., Jacobs R. (2020). Livedo reticularis as a presenting sign of severe acute respiratory syndrome coronavirus 2 infection. JAAD Case Rep..

[17] Steadmon M.J., Riley K.N. (2013). Erythema ab igne: A comeback story. J. Pediatr..

[18] Asilian A., Abtahi-Naeini B., Pourazizi M., Rakhshanpour M. (2015). Rapid Onset of Bullous Erythema Ab Igne: A Case Report of Atypical Presentation. Indian J. Dermatol..

[19] Kim R.H., Brinster N.K. (2020). Practical Direct Immunofluorescence. Am. J. Dermatopathol..

[20] Miller K., Hunt R., Chu J., Meehan S., Stein J. (2011). Erythema ab igne. Dermatol. Online J..

[21] Schieke S.M., Schroeder P., Krutmann J. (2003). Cutaneous effects of infrared radiation: From clinical observations to molecular response mechanisms. Photodermatol. Photoimmunol. Photomed..

[22] Hu C., Yang J., Qi Z., Wu H., Wang B., Zou F., Mei H., Liu J., Wang W., Liu Q. (2022). Heat shock proteins: Biological functions, pathological roles, and therapeutic opportunities. MedComm.

[23] Calapre L., Gray E.S., Ziman M. (2013). Heat stress: A risk factor for skin carcinogenesis. Cancer Lett..

[24] Shellman Y.G., Howe W.R., Miller L.A., Goldstein N.B., Pacheco T.R., Mahajan R.L., LaRue S.M., Norris D.A. (2008). Hyperthermia induces endoplasmic reticulum-mediated apoptosis in melanoma and non-melanoma skin cancer cells. J. Investig. Dermatol..

[25] Park H.S., Lee J.S., Huh S.H., Seo J.S., Choi E.J. (2001). Hsp72 functions as a natural inhibitory protein of c-Jun N-terminal kinase. EMBO J..

[26] Daneshvar E., Seraji S., Kamyab-Hesari K., Ehsani A.H., Hanifnia A.R., Razavi Z. (2020). Basal cell carcinoma associated with erythema ab igne. Dermatol. Online J..

[27] Bazaliński D., Przybek-Mita J., Barańska B., Więch P. (2017). Marjolin’s ulcer in chronic wounds—Review of available literature. Contemp. Oncol..

[28] Smith T., Nambudiri V.E. (2018). Erythema ab igne. Cleve Clin. J. Med..

[29] Sahl W.J., Taira J.W. (1992). Erythema ab igne: Treatment with 5-fluorouracil cream. J. Am. Acad. Dermatol..

[30] Patel G.K., Goodwin R., Chawla M., Laidler P., Price P.E., Finlay A.Y., Motley R.J. (2006). Imiquimod 5% cream monotherapy for cutaneous squamous cell carcinoma in situ (Bowen’s disease): A randomized, double-blind, placebo-controlled trial. J. Am. Acad. Dermatol..

[31] Kim H.W., Kim E.J., Park H.C., Ko J.Y., Ro Y.S., Kim J.E. (2013). Erythema ab igne successfully treated with low fluenced 1064-nm Q-switched Neodymium-Doped Yttrium Aluminum Garnet laser. J. Cosmet. Laser Ther..

[32] Wipf A.J., Brown M.R. (2022). Malignant transformation of erythema ab igne. JAAD Case Rep..

[33] Sachedina A., Todd N. (2020). Dysmenorrhea, Endometriosis and Chronic Pelvic Pain in Adolescents. J. Clin. Res. Pediatr. Endocrinol..

[34] Chapron C., Borghese B., Streuli I., de Ziegler D. (2011). Markers of adult endometriosis detectable in adolescence. J. Pediatr. Adolesc. Gynecol..

[35] Harel Z. (2012). Dysmenorrhea in adolescents and young adults: An update on pharmacological treatments and management strategies. Expert. Opin. Pharmacother..

[36] Stratton P., Berkley K.J. (2011). Chronic pelvic pain and endometriosis: Translational evidence of the relationship and implications. Hum. Reprod. Update.

[37] Sanfilippo J., Erb T. (2008). Evaluation and management of dysmenorrhea in adolescents. Clin. Obstet. Gynecol..

[38] Stuparich M.A., Donnellan N.M., Sanfilippo J.S. (2017). Endometriosis in the Adolescent Patient. Semin. Reprod. Med..

[39] Gierthmühlen J., Baron R. (2016). Neuropathic pain. Semin. Neurol..

[40] Shrikhande A.A. (2020). The consideration of endometriosis in women with persistent gastrointestinal symptoms and a novel neuromusculoskeletal treatment approach. Arch. Gastroenterol. Res..

[41] Yanchun L., Yunhe Z., Meng X., Shuqin C., Qingtang Z., Shuzhong Y. (2019). Removal of an endometrioma passing through the left greater sciatic foramen using a concomitant laparoscopic and transgluteal approach: Case report. BMC Womens Health.

[42] Steinberg J.A., Gonda D.D., Muller K., Ciacci J.D. (2014). Endometriosis of the conus medullaris causing cyclic radiculopathy. J. Neurosurg. Spine.

[43] Morotti M., Vincent K., Brawn J., Zondervan K.T., Becker C.M. (2014). Peripheral changes in endometriosis-associated pain. Hum. Reprod. Update.

[44] Wu J., Xie H., Yao S., Liang Y. (2017). Macrophage and nerve interaction in endometriosis. J. Neuroinflammation.

[45] Phan V.T., Stratton P., Tandon H.K., Sinaii N., Aredo J.V., Karp B.I., Merideth M.A., Shah J.P. (2021). Widespread myofascial dysfunction and sensitisation in women with endometriosis-associated chronic pelvic pain: A cross-sectional study. Eur. J. Pain.

[46] Agarwal S.K., Chapron C., Giudice L.C., Laufer M.R., Leyland N., Missmer S.A., Singh S.S., Taylor H.S. (2019). Clinical diagnosis of endometriosis: A call to action. Am. J. Obstet. Gynecol..

[47] Speer L.M., Mushkbar S., Erbele T. (2016). Chronic Pelvic Pain in Women. Am. Fam. Physician.

[48] Demetriou L., Krassowski M., Abreu Mendes P., Garbutt K., Vitonis A.F., Wilkins E., Coxon L., Arendt-Nielsen L., Aziz Q., Birch J. (2023). Clinical profiling of specific diagnostic subgroups of women with chronic pelvic pain. Front. Reprod. Health.

[49] Gholiof M., Adamson-De Luca E., Foster W.G., Leyland N.A., Bridge-Cook P., Leonardi M., Wessels J.M. (2023). Prevalence of Use and Perceived Effectiveness of Medical, Surgical, and Alternative Therapies for Endometriosis Pain in Canadians. J. Obstet. Gynaecol. Can..

[50] Norman M., Razmpour O., Olsen J.M. (2021). Women’s Use of Self-Care Interventions for Endometriosis Pain in the United States. Nurs. Womens Health.

[51] Steiner G.Z., Barry R.J., Wassink K., De Blasio F.M., Fogarty J.S., Cave A.E., Love S., Armour M. (2020). Neuronal Correlates of Cognitive Control Are Altered in Women with Endometriosis and Chronic Pelvic Pain. Front. Syst. Neurosci..

[52] Zhao S., Wu W., Kang R., Wang X. (2021). Significant Increase in Depression in Women with Primary Dysmenorrhea: A Systematic Review and Cumulative Analysis. Front. Psychiatry.

[53] Szypłowska M., Tarkowski R., Kułak K. (2023). The impact of endometriosis on depressive and anxiety symptoms and quality of life: A systematic review. Front. Public Health.

[54] Maulitz L., Stickeler E., Stickel S., Habel U., Tchaikovski S.N., Chechko N. (2022). Endometriosis, psychiatric comorbidities and neuroimaging: Estimating the odds of an endometriosis brain. Front. Neuroendocrinol..

[55] Meltzer-Brody S., Leserman J. (2011). Psychiatric comorbidity in women with chronic pelvic pain. CNS Spectr..

[56] Arévalo-Martínez A., Moreno-Manso J.M., García-Baamonde M.E., Blázquez-Alonso M., Cantillo-Cordero P. (2022). Psychopathological and neuropsychological disorders associated with chronic primary visceral pain: Systematic review. Front. Psychol..

[57] Nagy H., Carlson K., Khan M.A.B. (2025). Dysmenorrhea.

[58] Akin M.D., Weingand K.W., Hengehold D.A., Goodale M.B., Hinkle R.T., Smith R.P. (2001). Continuous low-level topical heat in the treatment of dysmenorrhea. Obstet. Gynecol..

[59] Akin M., Price W., Rodriguez G., Erasala G., Hurley G., Smith R.P. (2004). Continuous, low-level, topical heat wrap therapy as compared to acetaminophen for primary dysmenorrhea. J. Reprod. Med..

[60] Putra I.B., Jusuf N.K., Dewi N.K. (2022). Skin Changes and Safety Profile of Topical Products During Pregnancy. J. Clin. Aesthet. Dermatol..

